# Exploiting DNA Endonucleases to Advance Mechanisms of DNA Repair

**DOI:** 10.3390/biology10060530

**Published:** 2021-06-14

**Authors:** Marlo K. Thompson, Robert W. Sobol, Aishwarya Prakash

**Affiliations:** 1 Mitchell Cancer Institute, University of South Alabama Health, Mobile, AL 36604, USA; mkt1921@jagmail.southalabama.edu (M.K.T.); rwsobol@southalabama.edu (R.W.S.); 2 Department of Biochemistry and Molecular Biology, University of South Alabama, Mobile, AL 36688, USA; 3 Department of Pharmacology, University of South Alabama, Mobile, AL 36688, USA

**Keywords:** CRISPR, gene editing, base excision repair, mismatch repair, homologous recombination, non-homologous end-joining, microhomology-mediated end-joining

## Abstract

**Simple Summary:**

Approaches to manipulate the genome of an organism, both selectively and accurately, are powerful techniques that can influence research practice, with extensive application to agriculture and medicine, including the ability to impact disease risk or onset. In this review article, we highlight the advances, made over several decades, on the procedures and capacities to facilitate genome editing, manifest with the discovery, characterization, and optimization of the mechanism for processing of clustered regularly interspaced short palindromic repeat sequences (CRISPR). The editing molecules in the CRISPR gene modification system behave as molecular scissors, cutting DNA at specific genetic locations. First identified as a natural defense mechanism in bacteria, the CRISPR system has now been extensively modified for use in almost all mammalian cells. In describing each CRISPR mechanistic class, we acknowledge the differences and positive attributes each class has to offer to support editing that allows the creation of gene knockouts, knock-ins, gene tagging, insertions, deletions, and point mutations. Further, we discuss how these editing strategies have shaped the field of DNA repair. Specifically, we provide examples of the utility of CRISPR approaches in furthering our understanding of two of the major DNA repair pathways, namely mismatch repair and base excision repair.

**Abstract:**

The earliest methods of genome editing, such as zinc-finger nucleases (ZFN) and transcription activator-like effector nucleases (TALENs), utilize customizable DNA-binding motifs to target the genome at specific loci. While these approaches provided sequence-specific gene-editing capacity, the laborious process of designing and synthesizing recombinant nucleases to recognize a specific target sequence, combined with limited target choices and poor editing efficiency, ultimately minimized the broad utility of these systems. The discovery of clustered regularly interspaced short palindromic repeat sequences (CRISPR) in *Escherichia coli* dates to 1987, yet it was another 20 years before CRISPR and the CRISPR-associated (Cas) proteins were identified as part of the microbial adaptive immune system, by targeting phage DNA, to fight bacteriophage reinfection. By 2013, CRISPR/Cas9 systems had been engineered to allow gene editing in mammalian cells. The ease of design, low cytotoxicity, and increased efficiency have made CRISPR/Cas9 and its related systems the designer nucleases of choice for many. In this review, we discuss the various CRISPR systems and their broad utility in genome manipulation. We will explore how CRISPR-controlled modifications have advanced our understanding of the mechanisms of genome stability, using the modulation of DNA repair genes as examples.

## 1. Introduction

**Figure 1:**
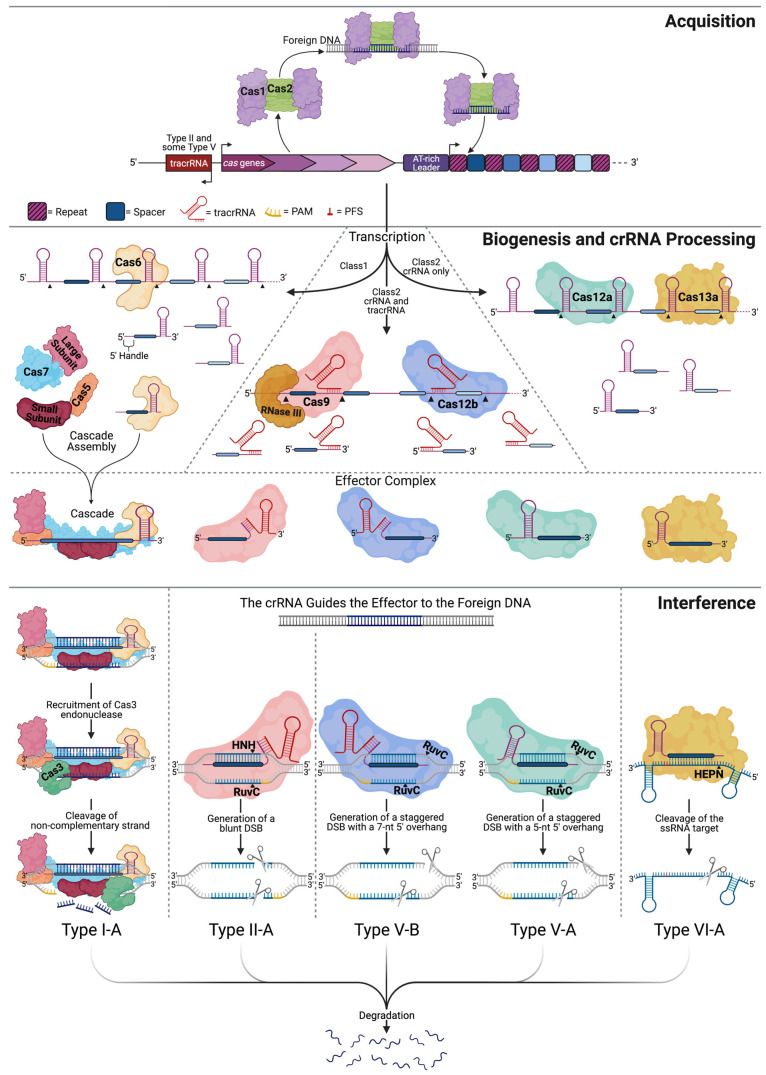
Comparison between Class I and Class II CRISPR immunity pathways. CRISPR immunity takes place in three general steps: acquisition, biogenesis, and interference. Type I-A, II-A, V-B, V-A, and VI-A are examples to illustrate some of the differences between each CRISPR type. In the majority of CRISPR systems, the acquisition is carried out by the Cas1-Cas2 complex, which locates a protospacer within the foreign DNA, excises it, and incorporates the new spacer into the CRISPR array. Biogenesis occurs upon recognition of a previous invader, producing the CRISPR-associated proteins, a long unprocessed CRISPR RNA (crRNA), and any additional RNAs, such as the trans-activating CRISPR RNA (tracrRNA). Processing may occur by the effector protein alone (Cas12a/13a), the effector protein with additional factors (Cas9/12b), or by a subunit of the effector complex (Cas6). Interference begins with the fully assembled effector complex scanning for a target sequence complementary to the spacer of the crRNA and protospacer adjacent motif (PAM) (yellow). Relative to the protospacer on the noncomplementary strand, Cascade and Cas12a/b look for a 5′ PAM, whereas Cas9 looks for a 3′ PAM. Cascade recruits Cas3 and cleaves the exposed non-complementary strand, manipulating the target strand into an ssDNA molecule. Similarly, Cas9 and Cas12a/b locate a target sequence followed by the invasion of the DNA double helix by the crRNA. Cas9 forms a blunt double-stranded break (DSB) within the complementary region, where the HNH domain cleaves the target strand and the RuvC-like domain cleaves the non-target strand. Cas12a and Cas12b form staggered DSBs, cleaving within and 5′ of the complementary region on the non-target and target strands, respectively. In the case of RNA targeting Cas13a, the cleavage location is determined by the nucleotide 3′ of the protospacer, known as the protospacer flanking sequence (PFS) (red). The Cas13a HEPN domain cleaves the target 5′ of the complementary region. CRISPR interference triggers the degradation of foreign nucleic acids.

CRISPR (Clustered Regularly Interspersed Short Palindromic Repeats) loci have been found in 50% of currently sequenced bacterial genomes, in 90% of the sequenced archaeal genomes, and have been identified in several genomes of some larger phages [1,2,3]. The locus consists of genes for CRISPR-associated proteins (Cas proteins) followed by a CRISPR array, which includes a promoter, containing an AT-rich leader sequence, preceding a series of unique spacer sequences interspersed by direct repeats (Figure 1) [4,5]. The CRISPR array is a detailed genetic record of prior infections, with each ~20 nt spacer having been obtained directly from the genome of a foreign invader [6,7,8,9]. CRISPR-mediated immunity against an invader occurs in three steps: acquisition (adaptation), biogenesis, and interference [10,11]. During infection by an unencountered invader, the cell has enough time to recognize the presence of foreign nucleic acids and acquire a new spacer from the foreign genome and incorporate it into the CRISPR array. Detection of a previously encountered invader triggers transcription and processing of the CRISPR array. Once transcribed, the effector component of the CRISPR system recognizes and binds the CRISPR RNA (crRNA) that guides the effector to hydrolyze the foreign genome [10,11].

There are two classes of CRISPR-Cas systems, based on the architectures of their effector molecules, which are responsible for DNA/RNA binding and recognition [12,13]. The two classes are subdivided into types I-VI, based on the presence of signature proteins, and are further divided into 48 subtypes. Class I systems, comprising Types I, III, and IV, have elaborate organizations and utilize multi-subunit effector molecules (Figure 1). Class II systems, consisting of Type II, V, and VI, exploit single subunit effectors [12,13]. The ease of expressing a single subunit protein versus a multi-subunit effector lead to Class II systems that are more commonly implemented for genome editing. Steps in native CRISPR immunity will be described using Type I and Type II as examples for Class I and Class II systems, respectively. Figure 1 depicts the process for Types I, II, V, and VI. 

Once the cell recognizes the presence of a foreign invader, a protospacer element to be incorporated as a new spacer into the CRISPR array is identified, based on a 2–5 nt protospacer-adjacent motif (PAM). Without the PAM sequence, the foreign DNA cannot be incorporated or later targeted for cleavage during interference [14]. Cas1 and Cas2 are the most conserved proteins among CRISPR systems and appear to be the minimal machinery needed for acquisition [10,15]. Cas1 is a DNase capable of cleaving single-stranded (ss), double-stranded (ds), cruciform, and branched DNA, whereas Cas2 is an endoribonuclease with a preference for ssRNA [16,17]. The crystal structure of the Cas1-Cas2 complex (PDB: 5DQU) reveals that two Cas1 dimers are connected via a Cas2 dimer, as shown in Figure 1 [18]. Depicted in complex with a 23-base pair (bp) DNA duplex with 5′-(T)_6_ and 3′-(T)_10_ overhangs, Wang and colleagues discovered that the Cas1 dimers stabilize the ends of the protospacer. In contrast, the center segment is stabilized through charge-charge interactions between the phosphate backbone and the positively charged surface of the Cas2 protein [18]. The Cas1/2 orthologs and the distance between the Cas1 dimers in the Cas1-Cas2 complex determine the length of the acquired spacer. Once Cas1 cuts the DNA backbone to remove the protospacer, the integration of the new spacer into the CRISPR array is favored at the leader sequence-repeat interface [19]. To maintain the repeat-spacer architecture, each strand of the spacer is integrated into opposite sides of the proximal repeat [20]. The 5′ PAM nucleotide allows the new spacer to be properly oriented within the CRISPR array, incorporating the last nucleotide of the PAM sequence as the first nucleotide of the new spacer. By not including the entire PAM sequence, the cell is able to avoid self-targeting [20,21].

Transcription of the CRISPR array yields a single long RNA molecule that must be processed into smaller functional crRNA molecules [22]. The significant players in crRNA processing for several CRISPR types are depicted in Figure 1 [23]. In Type I systems, the unprocessed RNA naturally forms stem-loop structures within the repeats. The interactions in Type I systems between the RNA and the processing enzyme, Cas6, are both structural and sequence-specific [24]. Cas6 cleaves RNA within the repeats, leaving a 5′ handle and a 3′ stem-loop structure [25]. Once processed, the crRNA associates with the appropriate interference complex that will locate and destroy the foreign DNA. In Type II systems, a trans-activating CRISPR RNA (tracrRNA) partially hybridizes to the CRISPR repeat within the crRNA, forming a tracrRNA:crRNA hybrid that recruits Cas9 [23,26]. Cas9 binds the 3′ end of the tracrRNA, stabilizing the tracrRNA:pre-crRNA complex, allowing RNaseIII to cleave the pre-crRNA [23,26]. Once cleaved, the tracrRNA:crRNA remains complexed with Cas9 and is primed for interference.

**Figure 2:**
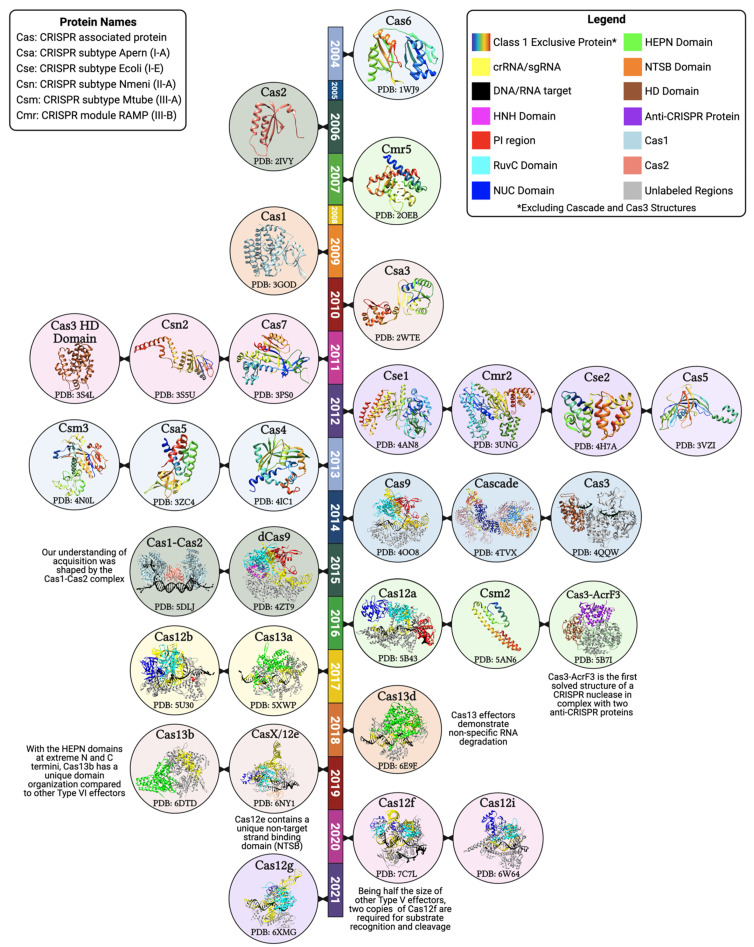
Timeline of the major CRISPR-associated protein structures. Structural biology has played a major role in understanding the organization of CRISPR proteins and their interactions with the target DNA, crRNA, and other associated proteins. The majority of CRISPR systems fall into Class I (rainbow-colored structures). To differentiate Cas1 and Cas2 within the Cas1-Cas2 acquisition complex, they are represented in solid colors that are maintained in their independent structures (see legend). The multi-subunit Class I effector, Cascade, is colored to differentiate each of the five proteins that make up the assembly: the large subunit (orange), small subunit (navy), Cas5 (blue), Cas6 (red), and Cas7 (pink). Starting in 2014 with the structure of Cas9, there is a shift in the proportion of solved structures belonging to Class II systems. Represented Class II structures are colored to illustrate the endonuclease domain organization. Additionally, in highlighting the nuclease domains, we can appreciate the bilobed (NUC and REC lobes) organization of Class II effectors. Protein data bank (PDB) IDs are included for each structure [18,30,31,35,48,49,50,51,52,53,54,55,56,57,58,59,60,61,62,63,64,65,66,67,68,69,70,71,72,73].

The broadest level of CRISPR classification is based on the effector complex that carries out the interference step. Class I and II systems employ a multi-subunit and single-subunit effector complex, respectively. In Type I systems, the multi-subunit effector, Cascade (CRISPR-associated complex for antiviral defense), contains five proteins: a small subunit, a large subunit, Cas5, Cas6, and Cas7 [27,28,29,30,31]. The crRNA guides the Cascade complex to the foreign DNA where Cascade binds and unwinds the target and recruits Cas3 to degrade the DNA [28]. The large subunit recognizes the PAM sequence at the 5′ end of the crRNA and recruits Cas3. Cas7 forms the crescent backbone along the crRNA and helps prepare the DNA for degradation. The small subunit aids in stabilizing the crRNA-target DNA complex, and Cas5 binds the 5′ handle of the crRNA, while Cas6 remains bound to the 3′ hairpin [28,32,33,34]. Upon Cascade binding to the target DNA, a conformational change allows the Cas3 HD domain (Figure 2, brown) to hydrolyze the noncomplementary strand backbone [10,35,36]. Manipulating the target strand into a ssDNA molecule permits its own degradation (Figure 1).

Type II systems have a more streamlined interference step, carried out by a single subunit effector, Cas9 [23,26]. The action of Cas9 takes place in a step-wise manner. The Cas9-tracrRNA:crRNA complex binds the foreign DNA, scans the molecule for a G-rich PAM motif (5′-NGG-3′), and looks for complementary base pairing between the target DNA and the 10-12 nt seed region at the 5′ end of the crRNA. Upon Cas9 binding to the PAM, the region upstream of the PAM partially unwinds, leading to the formation of an R-loop, allowing the crRNA to invade and base pair with the complementary sequence of the target DNA [14,26,37,38,39,40,41,42]. Together, the crRNA spacer and PAM determine the binding specificity of a CRISPR effector [14,26,38,43]. Mismatches between the crRNA, particularly the seed region, and the target DNA reduce efficient Cas9-induced cleavage [44,45]. Once the DNA is unwound, conformational changes allow the nuclease domains of Cas9 to cleave the target sequence ~3 bp upstream of the PAM sequence, introducing a double-strand break (DSB) with blunt ends [26,32,38,46,47].

## 2. Harnessing CRISPR for Genome Editing

The Type II Cas9 endonuclease has become the most characterized and implemented effector across CRISPR systems [12,74]. To exploit the genome editing power of Cas9, only two other factors need to be deployed: the crRNA and the tracrRNA. The tracrRNA:crRNA dual RNA guide has been further engineered into a single guide RNA (sgRNA) by linking the 5′ end of the tracrRNA with the 3′ end of the crRNA [26,46]. Many online and open-source tools exist to aid researchers in designing sgRNAs proximal to a PAM sequence to target their region of interest within the genome. Many of these tools have been gathered and organized by Barman et al. [75].

Several Cas orthologs have been identified, each displaying varied protein size, unique PAM consensus sequences, editing windows, spacer lengths, and other genome editing properties, allowing for increased diversity while selecting the Cas9 ortholog best suited for a particular experiment [12,74,76]. Modification of the PAM-interacting (PI) domain of Cas9 also enables alteration of PAM specificity [59]. Table 1 contains various Class II effector proteins, natural orthologs and engineered variants, with their respective unique features. Following selection of the effector protein and design of the sgRNA, delivery of the sgRNA into the cell must be considered [77,78]. This decision is based on multiple factors, including the application (in vitro, in vivo, or ex vivo), time frame, efficiency, delivery substrate (DNA, RNA, or protein), dose control, equipment available, and cost. An in-depth look at CRISPR delivery strategies is documented elsewhere [77,78].

## 3. Structural Properties of the CRISPR Effector Molecules

Three-dimensional structures of the effector molecules obtained using high-resolution methodologies such as X-ray crystallography, cryo-electron microscopy (cryo-EM), and nuclear magnetic resonance (NMR) have played a crucial role in our understanding of the function of the various Cas proteins (Figure 2). Cas9 adopts a bi-lobed, “jaw-like” shape containing an α-helical recognition (REC) lobe and a nuclease (NUC) lobe [59,111]. The REC lobe is the most variable region of Cas9 and is responsible for interacting with the sgRNA (Figure 2, yellow) and connecting the lobes through an arginine-rich bridge helix. The NUC lobe contains an HNH domain (Figure 2, magenta), RuvC-like domain (Figure 2, cyan), and the PI region on the C-terminal end (Figure 2, red) [59]. Once at the target sequence, the target DNA-sgRNA hybrid region of the R-loop becomes sandwiched between the lobes in a positively charged groove [59]. During the formation of the R-loop, the distortion of the double helix leads to a conformational change in Cas9 that places the HNH and RuvC-like catalytic sites adjacent to the target and displaced non-target strand, respectively [112]. The HNH and RuvC-like domains are responsible for cleaving the target (complementary) and non-target (noncomplementary) strand, respectively [46,113,114,115].

The critical difference between Type II, V, and VI, which all belong in Class II, is the domain architecture of the effector molecules. Type II effectors (Cas9) contain an HNH domain inside the RuvC-like domain. Their nuclease activity requires each domain to cut the target region to form a DSB [26,46]. In contrast, the Type V (Cas12) nucleases contain both a RuvC-like (Figure 2, magenta) and a NUC domain (Figure 2, dark blue), with the RuvC-like domain responsible for cleavage of both strands [63,97,116,117]. For many Cas12 nucleases, cleavage of the target and non-target strands occur independently, with the non-target strand being cleaved in a PAM and sequence-independent manner [66,118,119,120]. The result is a DSB with staggered ends (Figure 1), the length of which can vary between subtypes [66,95,97].

Lacking any DNase domains, Type VI effectors (Cas13) do not contain a RuvC-like domain, an HNH domain, or a NUC domain (Figure 2) [106]. Instead, Type VI effectors contain two higher eukaryotes and prokaryotes nucleotide (HEPN)-binding domains (Figure 2, green) for their RNA cleavage activity [106]. Once bound to the ssRNA target, a conformational change occurs within Cas13 that activates the cleavage site, relocating the HEPN active sites far from the target RNA [121]. The cleavage site activation enables Cas13 to cleave any nearby ssRNA non-specifically [67]. This effect could be an evolved mechanism to trigger cell death or induce dormancy during a severe infection [106,122]. Regions performing the nuclease activity of Class II effectors are highlighted in Figure 2.

## 4. Incorporating DNA Edits via Cas9 and DSB Repair

**Figure 3:**
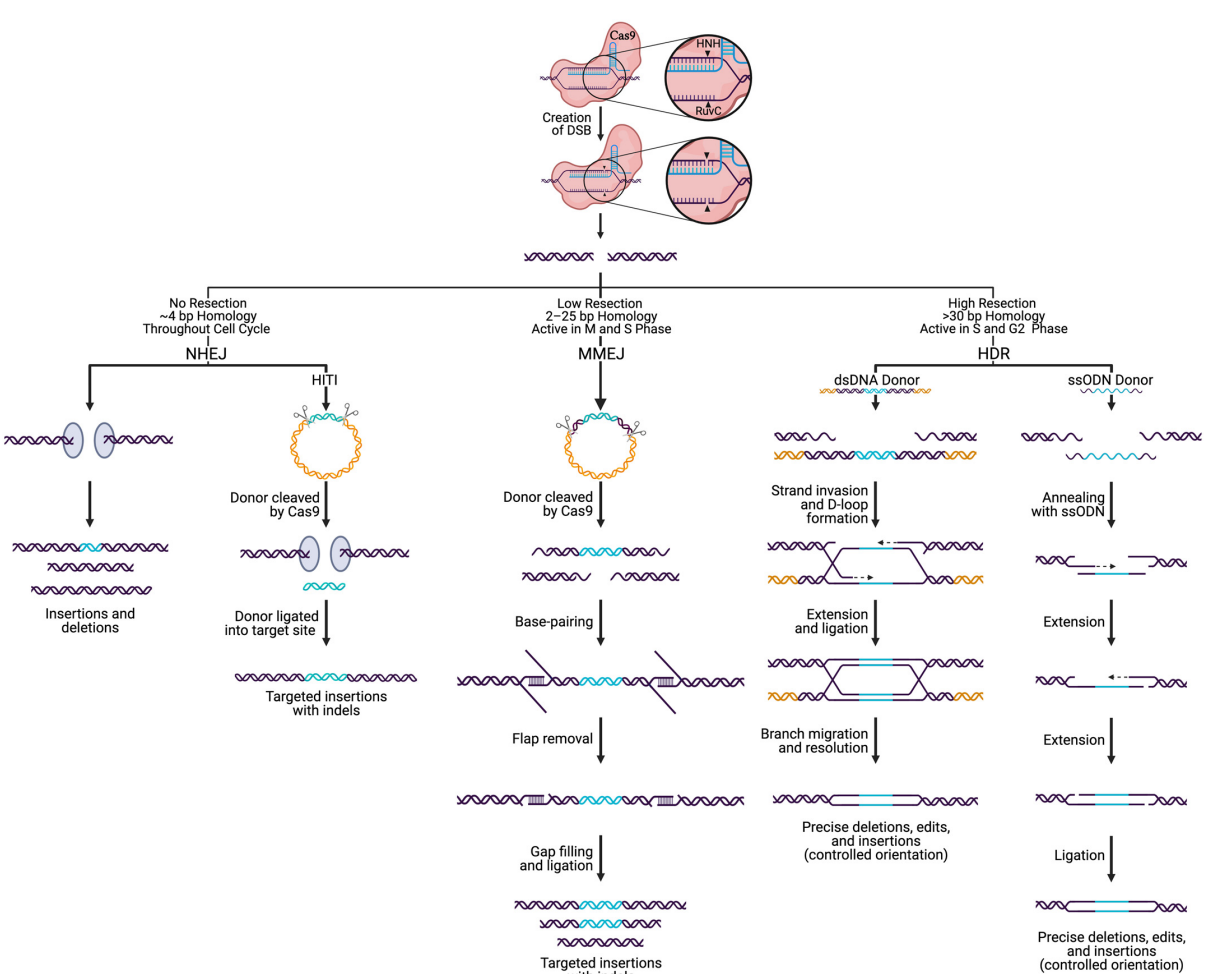
Pathways that repair SpCas9-directed DNA breaks. The pathway employed to repair Cas9 generated double-strand breaks (DSBs) is dependent on the repair factors present, level of resection, extent of homology, and cell cycle phase. If the ends of a DSB are immediately capped by Ku proteins, preventing resection, the nonhomologous end-joining pathway (NHEJ) initiates to ligate the DNA ends back together. NHEJ leads to uncontrolled insertions and deletions (indels), varying in size. Specific insertions can be incorporated through NHEJ via homologous independent targeted insertions (HITI) by supplying a double-stranded (ds) DNA donor flanked by Cas9 cleavage sites. During the M and S phase, when low levels of resection occur in the presence of 2–25 bp of homology, microhomology-mediated end-joining (MMEJ) takes place that results in small indels. During S and G2 phases, homology-directed repair (HDR) can achieve precise deletions, insertions, and edits, utilizing ss or ds donors.

DSBs are considered the most severe type of DNA damage, leading to genotoxicity if left unrepaired; thus cells have evolved multiple pathways to repair DSBs (Figure 3) [123,124,125,126,127,128,129]. Non-homologous end joining (NHEJ) and homology-directed repair (HDR) are the main avenues of repair for DSBs, accompanied by less common methods of repair such as microhomology-mediated end joining (MMEJ) [130,131,132]. Each pathway involves DSB recognition, end processing, polymerase activity, and finally, ligation. Aside from the cell cycle phase, pathway choice is dictated by the complexity of the DSB site (presence of lesions, sugar modifications, or base modifications) and the level of end-resection that takes place. For instance, shorter resections appear to favor MMEJ, while more extensive resection undergoes HDR [133].

NHEJ is the predominant DSB repair pathway in most eukaryotes, active throughout the cell cycle, with increased activity in the progression from G1 to G2/M [134,135,136,137,138,139,140]. NHEJ repairs DSBs through direct ligation of broken ends, either without a homologous template or with up to 4 bp of microhomology [141]. During NHEJ: (i) the Ku heterodimer recognizes and binds the broken ends, (ii) factors such as the DNA-dependent protein kinase catalytic subunit (DNA-PKcs) and nuclease Artemis are recruited to the site for processing, (iii) nucleotide addition by a polymerase can occur if needed, and (iv) the DNA-ligase IV complex performs ligation of the ends [141]. Often, the broken DNA ends undergo processing by exonucleolytic or endonucleolytic enzymes to expose regions of ≤4 nt microhomology, which leads to deletions of various sizes. It is worth noting that NHEJ can be used to delete entire genes by utilizing two sgRNAs designed to target each end of the gene [142,143,144]. On the other hand, the activity of DNA polymerase μ and λ can lead to a gain of nucleotides, resulting in insertions. Given the fast kinetics of repair, high efficiency, and high rate of indel formation, NHEJ has historically been exploited for targeting large deletions and gene knockout (KO) generation. More recently, knock-ins have been added to the repertoire of gene editing processes exploiting NHEJ through homology-independent targeted integration (HITI) [145,146]. HITI is an NHEJ-related approach that utilizes a donor fragment flanked by Cas9 cleavage sites [145,146]. Cas9 cleaves the donor sequence and the genomic target at the same time, triggering the NHEJ pathway and can result in ligation of the linear donor fragment into the target locus. HITI is also used to generate targeted edits and knock-ins in dividing and non-dividing cells by creating blunt end DSBs in the target and donor and has a higher efficiency than HDR, as NHEJ is more active in the cell [145,146].

In contrast to NHEJ, HDR requires a template sequence containing extensive homology, up to several hundred bases. Due to the need for such a template, HDR is less error-prone than NHEJ [147,148,149]. However, the activity of HDR is restricted to the late S and G2 phases of the cell cycle and is therefore limited to actively dividing cells [149,150,151]. It has been suggested that HDR constitutes roughly 15–20% of DSB repair during G2 [147,152]. The choice between NHEJ and HDR during the G2 cell cycle phase is attributed to factors present during the DNA end resection step [150]. For HDR to occur, one strand of the DSB is resected 5′ to 3′ to produce a terminal 3′-OH ssDNA tail that can intertwine with a homologous donor locus to prime for DNA synthesis by forming the displacement loop (D-loop) [153,154,155]. After strand synthesis begins, disruption of the D-loop may lead to the synthesis-dependent strand annealing (SDSA) pathway [155]. SDSA is a conservative method that is made up exclusively of non-crossover events. The newly synthesized strand becomes displaced from the template and returns to the processed end of the non-invading strand at the other DSB end. The non-invading strand is then elongated to fill the gap, forming a double Holliday junction (dHJ). The dHJ is resolved by nucleolytic action and has the potential for crossover events to occur [155,156,157]. Having originated from the same source, the cell favors the sister chromatid as a template, however, crossover events during HDR can be high-jacked to generate knock-ins, gene-tagging, and targeted gene editing [158,159,160]. In general, it is accepted that the process of HDR is slow, as compared to the more rapid mechanism of NHEJ (taking hours to complete HDR as compared to ~30 min for NHEJ). As such, enormous effort has been put forth to find techniques that increase HDR efficiency, such as cold shock and various small molecules, to inhibit or promote NHEJ and HDR, respectively [161,162,163,164]. More recently, single-strand oligodeoxynucleotide (ssODN) donors with short (~40 nt) homology arms have been shown to increase the efficiency of HDR, allowing the successful insertion of ~1.5 kb DNA fragments [165,166,167]. Using Cas9, previously difficult to transfect cell types (human embryonic stem cells and induced pluripotent stem cells) have now been rendered available for HDR-mediated editing [168,169].

MMEJ requires additional factors, including poly (ADP-ribose) polymerase 1 (PARP1), DNA Polθ, and 2–25 bp of microhomology upstream and downstream of the DSB [141,170]. MMEJ is most active in M and early S phases of the cell cycle, when HDR is inactive, and has been shown to have a 2–3x higher efficiency for knock-in than HDR [170,171]. A DSB with microhomologies allows annealing that can result in a deletion of the intervening sequence [172]. More in-depth reviews of each DSB repair pathway and methods used to promote individual pathways can be found elsewhere [133,135,141,147,148,149,155,173].

## 5. Cas System Modifications

Since the initial implementation of Cas9 from *Streptococcus pyogenes* (SpCas9) for genome editing, modifications have continuously been made to improve the system. Enhancements have been made by modifying the associated RNA scaffold, as well as the Cas protein [174]. Early on, a nuclear localization sequence was added to Cas9 to allow easier nuclear import and the crRNA and tracrRNA were fused to form the sgRNA. Additionally, the sgRNA was modified to increase the binding efficiency of Cas9 to the target DNA [174]. With its robust editing power, Cas9 was found to exhibit a high level of off-target effects [175]. DSBs at off-target sites would likely be repaired by NHEJ, resulting in indel formation and could potentially lead to unintended changes in gene expression and/or function. However, several independent groups discovered that shortening the 5′ end of the sgRNA by 1–3 bp resulted in a decrease in the formation of DSBs at off-target sites, while retaining sufficient activity at the target site [176,177]. SpCas9 limits the choice of the target site to the location proximal to the PAM sequence of SpCas9, 5′-NGG-3′. Fortunately, these limitations can be mitigated using natural Cas orthologs and engineered Cas variants (Table 1). When designing a gene editing project, in general there are numerous details to consider, including: the Cas protein to employ, the PAM sequence requirements, the nucleic acid target, the type of edit being introduced and importantly, the method of Cas9/gRNA delivery (plasmid transfection, viral transduction, transfection of RNA-protein complexes).

### 5.1. Cas Orthologs

The identification of multiple Cas orthologs ushered in a more sophisticated means for selecting the PAM sequence best suited to an ideal target site (Table 1). Alternative PAM sequences that occur less frequently throughout the genome may narrow possible targets, but it is also one way to further reduce off-target effects. For instance, *Neisseria meningitides* Cas9 (NmCas9) recognizes the PAM site 5′-NNNNGATT-3′, which occurs about 12 times less frequently in the human genome than the SpCas9 PAM site [80,88]. Across Cas9 orthologs, there is a diverse assortment of PAM sequences: 5′-NNGRRT-3′ (*Staphylococcus aureus*), 5′-NNNVRYM-3′ (*Campylobacter jejuni*), 5′-NNNNGATT-3′ (*Neisseria meningitides*), 5′-NAAAAC-3′ (*Treponema denticola*) [91,178,179]. To calculate the occurrence of PAM sites within the human genome GRCh38/hg38 assembly, we used Python 3.9.2 with Biopython 1.78 [180,181] to develop a useful script that is available here [182]. The frequency of PAM sites for the different Cas orthologs, compared to that of SpCas9, can be found in Table 1.

In contrast to the Cas9 orthologs that often recognize a G-rich PAM at the 3′ end of the protospacer, Cas12 proteins (Type V) identify a T-rich PAM sequence found at the 5′ end of the protospacer [95]. To further differentiate them from their Type II counterparts, Type V effectors generate a DSB with 5′ overhangs rather than a DSB with blunt ends [66,95,97]. These sticky ends can be utilized to properly orient donor DNA and improve the ligation between broken ends via NHEJ repair [95]. Additionally, Cas12 nucleases often have indiscriminate ssDNA cleaving activity in addition to the targeted dsDNA sites [183]. The non-target cleavage activity by Cas12 nucleases has been repurposed for highly sensitive detection of specific nucleic acid sequences [183]. The majority of Cas12 nucleases reported higher specificity than that of Cas9, with Cas12b being sensitive to any single base change within the spacer region [97]. Cas12a (Cpf1) is as effective as Cas9, with fewer harmful off-target effects [184,185,186]. As with Cas9 orthologs, Cas12 shows diversity within the PAM requirements (Table 1). Cas12j recognizes a relaxed PAM, 5′-TBN-3′ [104]. Cas12f (Cas14) recognizes a T-rich PAM in dsDNA (‘5-TTTR-3′/5′-TTAT-3’) and cleaves ssDNA without PAM specificity [101,102]. Rather than dsDNA, Cas12g cleaves ssRNA and ssDNA with no PAM requirement [98,100,101].

Cas13, the Type VI effector, is an RNA endonuclease that recognizes the nucleotides adjacent to the protospacer, known as the protospacer-flanking site (PFS). As with PAMs, the PFS can vary between each of the Cas13 orthologs. LshCas13a shows bias for a 5′-PS-H-3′, while BzCas13b recognizes a 5′-D-PS-NAN/NNA-3′ motif [187,188,189]. The double-sided PFS enhances the specificity of Cas13b. Alternatively, Cas13d does not have a PFS requirement, giving it less restriction in targeting RNA [110]. Cas13 systems have already proven to be a useful tool in RNA interference, identifying specific RNA-associated proteins, and as a diagnostic tool [190,191]. The SHERLOCK platform (now SHERLOCKv2) utilizes Cas13a/b homologs to simultaneously detect multiple transcripts, such as Dengue and Zika virus ssRNA as well as RNA from SARS-CoV-2 [192,193,194,195,196]. Similar platforms employing Cas13 include SHINE (Streamlined Highlighting of Infections to Navigate Epidemics), developed to detect SARS-CoV-2 RNA, and PAC-MAN (prophylactic antiviral CRISPR in human cells), designed to trigger viral inhibition [197,198]. Additionally, Cas13b loci encode for small accessory proteins including Csx27 (VI-B1 systems) and Csx28 (VI-B2 systems), each known to regulate Cas13b activity. Cas13b can be stimulated and repressed by Csx28 and Csx27, respectively, which opens the door to alternative methods for controlling the CRISPR system [109].

### 5.2. Cas Variants

Since DSBs induced by the Cas9 system are recognized as severe genomic damage by the cell, engineered Cas variants have been developed to reduce unintended DSBs (Table 1). Hyper-accurate Cas9, or HypaCas9, a variant containing substitutions in the REC domain (N692A/M694A/Q695A/H698A), retains enhanced on-target activity as compared to WT SpCas9 while showing slower cleavage of substrates containing mismatches [83,199]. Other engineered Cas9 variants, designed to increase specificity and decrease off-target activity, include SpCas9-HF1 (high-fidelity), eSpCas9 (enhanced specificity), and HiFi Cas9 (high-fidelity) [85,86,87]. Cas9 variants have also been designed to circumnavigate PAM restrictions (Table 1). The engineered variant xCas9-3.7 was designed to have the broadest range of PAM recognition. The added PAM flexibility of xCas9-3.7 comes with increased DNA specificity and decreased off-target activity compared to SpCas9 [81]. The *Francisella novicida* Cas9 E1369R/E1449H/R1556A (FnCas9 RHA) recognizes the shortest PAM, 5′-YG-3′ [92]. SpCas9 D1135E has reduced binding to its non-canonical PAM, 5′-NAG-3′, compared to the WT [90]. The Cas9 nickase (nCas9) was created by inactivating one of the two nuclease domains via a mutation in either the RuvC domain (D10A) or the HNH domain (H840A) [26,59]. The single-strand breaks (SSBs) generated by nCas9 are less deleterious to a cell as they are repaired by the high-fidelity base excision repair (BER) pathway [200,201,202,203]. Utilizing nCas9 with two sgRNAs can increase the specificity of Cas9 and generate a DSB at the target site [204,205]. Catalytically dead Cas9 (dCas9) was developed by inactivating both nuclease domains (D10A/H840A or D31A/N891A) and yields an RNA-guided DNA-binding protein [26,46,206]. While dCas9 is unable to cut dsDNA, it retains the ability to open dsDNA in a PAM-dependent manner to allow the sgRNA to promote complementary pairing. Interestingly, pairing dCas9 with a FokI nuclease enhanced the cleavage efficiency of the target [207,208]. By tagging dCas9 with EGFP, researchers can visualize specific genomic loci. dCas9-EGFP has enabled the investigation of chromosome reorganization during cell division, as well as monitoring telomere dynamics [174]. dCas9 is often paired with effector domains to indirectly edit the genome, and more recently to create targeted single-base edits without a template, as discussed further below.

To gain spatiotemporal control over the expression of Cas9, split and inducible variants have been generated [209]. Split-Cas9 is divided into two or more fragments that can dimerize/multimerize into the functional form autonomously or with the use of adaptors. It can be difficult to insert certain full-length Cas9 constructs and sgRNA(s) into a size limited plasmid, such as the adeno-associated virus (AAV) vector, where the packing limit is based on the size of the parent AAV genome. By packaging each fragment separately, Split-Cas9 allows the incorporation of additional regulatory elements, sgRNAs, or protein tags [209]. In the split systems using adaptors, the dimerization can be controlled using light irradiation, small molecules, or temperature. Blue light-inducible Cas9 can be produced by inserting a blue light-excited LOV domain between F478 and E479. Alternatively, a blue light-inducible split Cas9 was generated using two fragments, Cas9 (2-713)-pMag and nMag-Cas9 (714-1368) [210,211]. Rapamycin can be used to control the nuclear localization of split Cas9 by inserting the FK506 binding protein (FKBP) and the FKBP12-rapamycin-binding (FRB) domain, as well as nuclear export signals (NES) and nuclear localization signals (NLS), resulting in Cas9 (1-573)-FRB-NES and NLS-FKBP-Cas9 (574-1368)-NLS [212].

The generation of dCas13 orthologs opened up similar doors in the RNA world as dCas9 did in the DNA world. dCas13 is an RNA-binding protein and is effective in binding targeted RNAs as well as in knockdown experiments, being as efficient as RNA interference (RNAi) with drastically reduced off-target effects and zero observed collateral activity in mammalian cells [108]. Further, dCas13 allows for visualization of specific RNA molecules, as well as regulation and editing on a post-transcriptional level [108,191,213].

### 5.3. Expression Modification Systems

Given its unique and highly specific DNA binding capacity in the absence of DNA hydrolysis, the dCas9 variant has also been used to modulate gene expression in two general ways: (i) dCas9 can sterically hinder regulatory elements, directly interfering with target gene expression or (ii) dCas9 can indirectly alter expression once fused with an effector domain that will interact with the regulatory elements of the target gene [204,206,214,215,216,217,218]. Fusions of dCas9 with DNMT3A or DNMT3a-DNMT3L repress transcription by increasing DNA methylation of target CpG motifs [219,220,221]. Alternatively, the dCas9-TET1 fusion increases transcription of a target by demethylating the target DNA regulatory element [222]. Moreover, fusions such as dCas9-p300 increase accessibility to genomic DNA by acting as targeted histone acetyltransferases [223].

CRISPR methods harnessing a Cas nuclease fused with transcriptional regulators to induce or repress gene transcription are known as CRISPR activation (CRISPRa) and interference (CRISPRi), respectively. CRISPRi most often pairs dCas9 to the KRAB (Kruppel-associated box of the Knox1 gene) repressor domain [206,215]. The KRAB domain represses gene transcription by altering the methylation patterns of targeted enhancer regions [224]. When comparing CRISPRi to RNAi or small-hairpin RNA (shRNA) methods, CRISPRi is found to be more efficient in reducing both gene expression and the resulting gene activity [214]. Limpitikul et al. demonstrated that CRISPRi could be used to functionally rescue iPSC-derived cardiomyocytes modeling long-QT syndrome by knocking down the mutant calmodulin2 (D130G) allele [225]. CRISPRa is composed of a more diverse set of systems. The first CRISPRa system fused dCas9 to a single VP64 activator domain [215,216]. The VP64 was later exchanged for the tripartite activator VP-p65-TRta, now known as the VPR CRISPRa system [226]. The SAM system utilizes a modified sgRNA scaffold with aptamers that generate additional binding sites for activator domains [227]. Alternatively, the addition of a long epitope tail to dCas9 can recruit multiple transcription activators. The SunTag (SUperNova explosion) system fused multiple copies of the VP16 activator domain to dCas9 [228]. Extensive review articles on CRISPRa/i can be found elsewhere [229,230,231,232,233,234].

### 5.4. Base and Prime Editors

**Figure 4:**
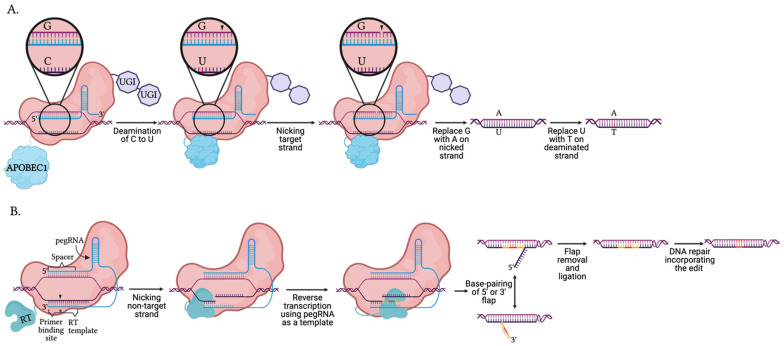
Mechanism of DNA base and prime editors using Cas9 nickase. (**A**) The illustrated generation 4 base editor (BE4) contains a nCas9 fused with two inhibitors uracil glycosylase inhibitors (UGI) and the cytosine deaminase, APOBEC1, capable of performing C to U transitions. APOBEC1 deaminates the target cytosine on the non-complementary strand, creating a uracil. The UGIs inhibit the repair machinery activated by the presence of the uracil base. nCas9 nicks the complementary strand and persuades the repair machinery to correct the non-deaminated strand, thereby promoting the incorporation of the edit. The final result is a C:G to T:A transition. (**B**). Prime editors contain a nCas9-reverse transcriptase (RT) fusion guided by an extended crRNA known as a primer editing guide RNA (pegRNA). The 5′ end of the pegRNA resembles a crRNA with a spacer region and a stem-loop. The 3′ end is extended to include a template containing the desired edit for the reverse transcriptase, and a 3′ primer binding site. The pegRNA guides nCas9 to the target DNA and forms Watson and Crick base-pairing at both the 5′ and 3′ ends. nCas9 nicks the PAM strand between the primer binding site and RT template. Using the primer binding site, the RT extends the PAM strand using the template containing the desired edit. Base pairing of the 3′ flap containing the edit leads to the removal of the 5′ flap. Through replication and DNA repair, the edit is incorporated into both strands.

To entirely avoid creating a DSB and the need for a donor template to generate point mutations, base editors (BEs) (Figure 4A) were created by fusing dCas9 or nCas9 to base modification enzymes. The catalytically deficient Cas (e.g., dCas9) binds the target DNA, enabling the sgRNA to hybridize with the target DNA. The sgRNA:DNA hybrid causes the PAM-containing region to be displaced and form a ssDNA R-loop [59,112]. The PAM-distal region within the R-loop is then accessible to the modification enzyme [112,118,235]. The first-generation base editors (BE1) utilized cytosine base editors (CBEs) to deaminate cytosine amine groups generating uridine (reads as thymine), leading to a C to T transition upon replication. However, the presence of the uracil base stimulates the BER pathway and could be removed by uracil DNA glycosylases (UDG), reverting the base pair back to the original G:C pairing [235]. BE2 added a UDG inhibitor (UGI) onto the C-terminal end of dCas9 to prolong the presence of uracil so as to prevent the reversion back to G:C and increase editing yield [235]. BE3 manipulated DNA repair machinery into correcting the non-edited strand to increase efficiency. Komor et al. fused the nCas9 to the cytosine deaminase, APOBEC1, and a UGI to prevent UDG from initiating BER and reverting the edit back to a cytosine [235]. To gauge editing efficiency, fluorescent probes have been employed [236]. To further increase the odds of the desired edit, the non-edited strand containing the guanine is then nicked by the nuclease [235,237]. Mutations within APOBEC1, such as W90Y/R126E/R132E, narrowed the editable window within the protospacer from positions 4-8 to 5-6 [238,239]. BE4 increased the lengths of existing linkers and added a second UGI at the C-terminus of BE3, increasing the editing efficiency and lowering the rate of indels [239]. Alternative Cas effectors have been substituted into base editing systems to control the PAM requirement and nucleic acid target [81,82,240]. 

The initial menu of base editors were limited until 2017 when Gaudelli et al. engineered a base editing adenine deaminase from a tRNA adenine deaminase (TadA) to convert adenines to inosines (read as guanines), allowing the conversion of A:T to G:C [238]. Now CBEs and ABEs (adenosine base editors) have been used in a variety of cell types and organisms to study point mutation mutagenesis and the biology associated with point mutation corrections [241,242,243,244,245,246]. By fusing the RNA-targeting dCas13 to ADAR2 (adenosine deaminase RNA specific) or the engineered cytidine deaminase, RNA editing (RESCUE; RNA editing for specific C-to-U exchange) can be reversible and temporally controlled [191,247]. Unfortunately, CBEs and ABEs remained limited to transitions, and not transversions, until prime editors (PEs) were introduced (Figure 4B). Prime editing targets insertions, deletions, or conversions of all 12 combinations of point mutations without a donor template or creating DNA DSBs [248]. First-generation prime editors (PE1) required just two elements: an nCas9-reverse transcriptase (RT) fusion protein and a primer editing guide RNA (pegRNA) [248]. The pegRNA contains the spacer sequence complementary to the target, a primer binding site region (8–16 nt), and the sequence containing the new genetic information to be introduced into the target. The pegRNA guides the Cas complex to the target, where the primer binding site binds the non-target strand. The RT then directly copies the genetic information from the pegRNA to the target strand [248].

However, the new capabilities in directed editing did not arrive without limitations. Off-target edits remained detectable using PE1 [248]. Thus, PEs have undergone an evolution of enhancements similar to that of BEs. PE2 utilized an engineered RT (M-MLV RT D200N/L603W/T330P/T306K/W313F), generating over a 5-fold increase in editing efficiency. PE3s brought the addition of a standard guide RNA to encourage nCas9 to nick the non-edited strand and keep the edit, as was done with BEs. Intriguingly, Anzalone et al. discovered PE3 is more efficient when the additional sgRNA matches the edited strand [248]. The rate of indels is partially contributed by the possibility that RT may extend into and thus insert part of the pegRNA scaffold. However, PEs remain limited to creating small indels. PE systems are unable to insert large segments of DNA, such as fluorescent tags, or target large deletions seen with previous Cas systems. Further shortcomings of the system include the large size of the fusion protein and the potential for random incorporation of cDNAs by the RT [248]. More in-depth reviews on BEs and PEs can be found elsewhere [249,250,251,252,253,254,255].

## 6. Mechanistic Advancement of DNA Repair Processes by CRISPR-Controlled Modifications

The rapid increase in the utility and broad availability of various CRISPR technologies has enabled an increased understanding of many cellular processes. The DNA repair community has benefitted greatly from the use of gene editing systems, which have contributed to our current understanding of repair mechanisms. Below, we will provide examples to highlight the advancement in knowledge of DNA mismatch repair (MMR) and BER.

### 6.1. Contribution of Gene Editing Technologies to MMR Biology

MMR is an essential post-replicative DNA repair mechanism that corrects base mismatches and replication slippage errors that have escaped proofreading by the replicative DNA polymerases [256,257,258]. As was eloquently described by Dr. Paul Modrich in his 

Nobel lecture (2015 Chemistry Nobel Laureate, for MMR, [259]), recognition of the mismatch and its subsequent repair, requires the concerted action of several players. First, the mismatch is recognized by the *E.coli* MutS protein followed by the recruitment of *E. coli* MutL. In *E. coli*, the endonuclease, MutH, cleaves the unmethylated nascent DNA strand to allow entry of exonuclease I that excises the mismatch-containing DNA strand. The resulting gap is filled by the DNA polymerase III holoenzyme and the nick is sealed by DNA ligase [259]. Along with UvrD (DNA helicase II) and single-stranded DNA binding protein, these components were sufficient for the in vitro reconstitution of MMR. Eukaryotic orthologs of the *E. coli* MMR machinery have since been identified and our current understanding of human MMR has been sculpted by contributions from several groups that are reviewed extensively elsewhere [259,260,261,262,263]. MMR also plays an important role in tumor suppression by not only repairing mismatched bases formed during DNA replication but also by inducing apoptosis to eliminate cells carrying modified DNA bases, such as *O*^6^-methylguanine [264,265,266]. In early seminal work looking at genome-wide editing using CRISPR/Cas9, a library of >73,000 sgRNAs that targeted over 7000 human genes was used to screen for MMR deficiency in the presence of 6-thioguanine (6-TG). In this resistance screen, the four primary components of the MMR pathway, MSH2, MSH6 (MutS homologs that form the MutSα complex), MLH1, and PMS2 (that form the MutLα complex) emerged as being resistant to treatment with 6-TG, one of the many hallmarks of MMR deficiency [267,268,269]. These large-scale screens are designed to test the efficacy and specificity of sgRNAs, providing us with the gene-editing tools needed to perform knockout studies with ease. In a recent report, a Cas9-mediated approach was used to generate a knockout of the ATP-dependent chromatin remodeling protein, SMARCAD1, resulting in a resistance phenotype to the alkylating agent N-methyl-N-nitrosourea. The SMARCAD1 protein is a known interacting partner of MSH2, part of the MutSα mismatch recognition complex. This report presents a new role for SMARCAD1 in MMR-dependent apoptosis in human cells by playing a part in the recruitment of the MutLα complex to MutSα [270].

Another hallmark of aberrant MMR is increased microsatellite instability (MSI), which includes insertion and/or deletion mutations that occur at DNA repeat sequences known as microsatellites. Increased MSI is associated with many cancers, including colorectal, endometrial, gastric, and ovarian cancers [271]. MLH1 inactivation via CRISPR/Cas9 knockout revealed an MMR deficiency with increased levels of MSI and elevated mutations that activate gene enhancers in a CRC model cell line [272]. Further, a large CRISPR/Cas9 screening endeavor led to the identification of a synthetic lethal relationship between the Werner syndrome helicase, WRN, and cancers displaying high MSI that will potentially lead to a future therapeutically relevant target [273,274].

Gene variants that result in pathogenic (i.e., disease-causing) mutations in one of the four primary MMR genes, *MSH2*, *MSH6*, *MLH1*, and *PMS2*, result in an autosomal dominant hereditary cancer condition known as Lynch syndrome (LS), further underscoring the clinical relevance of intact MMR in the cell [275,276,277]. LS patients and families have a high lifetime risk for various cancers, including an 80% risk for colorectal cancer, a 60% risk for endometrial cancer, and a lower risk for gastric, biliary tract, brain, and other cancers [275,278,279,280]. The term Lynch-like syndrome (LLS) has been ascribed to patients with MMR-deficient tumors that appear to have intact expression of the four canonical MMR genes, implicating the likely involvement of other factors in genome maintenance involving the MMR pathway. In a recent study, CRISPR/Cas9 knockout of *MCM8* revealed increased MSI and mutational signatures consistent with defective DNA repair, implicating a role for MCM8 during the MMR process [281].

In the United States, LS remains underdiagnosed and currently relies on genetic testing to confirm the presence of pathogenic mutations [278,282]. The diagnosis of LS is complicated by the identification of variants of uncertain significance or VUSs, where there is a lack of functional data regarding the particular variant. A VUS result is frustrating for both patients and healthcare providers alike since routine LS medical management practices are hindered. We and others have used in vitro techniques to address the issue of variant pathogenicity to assist with the reclassification of VUSs in MMR genes [283,284,285,286,287,288,289,290]. However, current common laboratory tests to determine pathogenicity are limited because they do not recapitulate the LS genotype. As such, utilizing sophisticated mutation analysis tools, offered by the various CRISPR strategies in engineered cell/tissue model systems, provides us with a platform to better understand how mutagenesis shapes tumor progression and cellular resistance to apoptosis. As an example of this, CRISPR/Cas9 tools were used to introduce several *MSH2* variants into the endogenous *MSH2* locus to characterize their functional consequence on MMR, DNA damage response, and ability to repair DNA microsatellite sequences at known endogenous loci [291]. Four of the ten variants scrutinized displayed strong evidence for being pathogenic, akin to the cells lacking MSH2 expression, while four others appeared to be benign with no effect on protein function [291]. The far-reaching impact of this data, when combined with future/existing co-segregation analyses, provides powerful diagnostic information for LS-families. In another study, genome editing via CRISPR/Cas9 was performed to introduce LS-relevant *MSH2* G674 mutations into HeLa cells. The resulting mutant cell lines exhibited MMR-defective phenotypes, displaying tolerance to alkylating agents and elevated mutation frequencies, characteristic of pathogenic mutations [292].

### 6.2. Advances in BER Biology by Exploiting Gene Editing Technologies

As we discuss above, CRISPR/Cas-based gene-editing systems have been enhanced and modified by employing various BER enzymes [203,293], made possible due to decades of elegant biochemical analysis of the enzymes in the BER pathway. Overall, BER, and the parallel sub-pathway single-stand break repair (SSBR), are both critical DNA repair pathways that are found in all species and are deemed essential for the resolution of base lesions and DNA single-strand breaks in both the nuclear and mitochondrial genomes. Extensive reviews on BER and SSBR are, of course, available [201,294,295,296,297,298,299,300,301,302]. As described in an elegant summary by Wilson and Kunkel, BER enzymes pass the resulting enzymatic products of each reaction of the repair mechanism to the next enzyme in the pathway [303], thereby avoiding the accumulation of genome destabilizing BER intermediates [304]. Given that these BER and SSBR enzymes function consecutively, it is suggested that the cellular expression level of each may be coordinated since over-expression is found to be detrimental [305,306,307,308]. Although the biochemistry of BER is well defined, it is understood that the formation of BER/SSBR complexes at genomic sites of base damage and DNA single-strand breaks requires additional layers of regulation and coordination due to the complexities of lesion access when DNA is within chromatin [202,309,310,311]. As such, the utility of such CRISPR/Cas systems for gene knockout, gene editing (point mutations), and gene tagging to clarify the biology of BER/SSBR in human systems is paramount.

For example, targeted CRISPR screens, with a focus on select DNA repair genes, have uncovered a role for BER in the response to cellular treatment with platinum drugs such as cisplatin [312]. Here, Lans et al. developed and validated a small 75-gene sgRNA library, specific for DNA repair genes across the various DNA repair pathways, with an emphasis on genes not likely to be lethal once deleted [312]. Cells depleted of each of the 75 DNA repair gene products were then evaluated for the cellular response to cisplatin. In addition to the expected group of genes that play a role in cisplatin-induced DNA repair, several BER genes were found to provide cellular protection against cisplatin-induced cell death, including POLB, XRCC1, and POLL. These studies then discovered that cisplatin leads to the induction of reactive oxygen species in cells, portending the requirement for BER [312]. Using a different but targeted set of sgRNAs, Nuyts et al. evaluated select DNA repair gene KOs for a role in radio-sensitization [313], uncovering a role for several BER genes in response to radiation in a subset of head and neck squamous carcinoma cell lines [313].

Generally, these overlapping pathways (BER and SSBR) can be broken into functional steps, as we have described previously [314]: (i) Lesion recognition & strand scission by the lesion removal enzymes (DNA glycosylases) and the primary BER endonuclease, APE1 or the alternate isoform APE2; (ii) PARP1 and PARP2 activation and poly(ADP-ribose) (PAR) synthesis to trigger chromatin and histone reorganization; (iii) DNA gap tailoring promoted by the scaffold protein XRCC1, helping to recruit DNA polymerase beta (Polβ), DNA Ligase III and APTX, with PAR-dependent recruitment of APLF and break-dependent recruitment of PNKP. Repair is then completed following DNA synthesis & ligation, conducted primarily by Polβ and DNA ligases I and III, together with the scaffold protein XRCC1. Finally, (iv) PAR molecules are degraded by PARG, ARH3, and TARG1, promoting chromatin reorganization and completion of repair. BER and SSBR are generally considered to function throughout the cell cycle and across the genome, although the regulatory factors involved are likely varied depending on chromatin status [202]. An example of such a factor, ALC1 (CHD1L), was recently uncovered, using targeted CRISPR screens, to play a role in both the cellular response to base damage (such as that induced by the alkylating agent methyl methane sulfonate, MMS) and the response to PARP1 inhibitors [315,316,317,318]. This is consistent with the reported role of ALC1 as an SNF2 ATPase chromatin remodeler activated by poly (ADP-ribose) [319,320]. To follow, we highlight each step in the BER/SSBR pathway and how CRISPR systems have helped advance or uncover the role of these pathways in cellular systems.

#### 6.2.1. BER/SSBR Lesion Recognition & Strand Scission

The first step in BER is attributed to DNA glycosylases, a family of 11 human genes that encode at least 14 different enzyme isoforms further regulated by variations in post-translational modifications or base editing [256,321,322]. These initiators of BER function by removing small base modifications such as 8-oxo-dG, inosine, and deoxyuracil, among many other base lesions that arise from both endogenous metabolites and exogenous (environmental) exposures [321]. Loss or inhibition of DNA glycosylase enzyme activity is mutagenic. As highlighted over 20 years ago by Thomas Lindahl (2015 Chemistry Nobel Laureate, for BER [323]), glycosylases are anti-mutator enzymes that suppress spontaneous and induced mutagenesis in human cells [324,325].

Recently, unique mutational signatures have been identified in human cancer [326]. A follow-up study set out to determine if these mutational signatures may be the result of unique DNA repair genes or pathway deficiencies [327]. DNA repair genes were deleted using the CRISPR/cas9 system in human colon organoids to identify DNA repair pathways and genes that may yield unique mutational signatures that correspond to those found in human cancers. One signature originally identified in human breast cancer, referred to as Signature 30, was found to be similar to that found following *NTHL1* gene deletion, suggestive that “signature 30” may arise from *NTHL1* gene mutations. The *NTHL1* gene product is a human DNA glycosylase with homology to *E. coli* endonuclease III [328,329] that is specific for the repair of oxidative base lesions such as 5-hydroxycytosine, thymine glycol, 5-hydroxy-6-hydrothymine, 5,6-dihydroxycytosine, and 5-hydroxyuracil [330], as well as 8-oxodG [331], with specificity for damage opposite a guanine base, summarized in the review by Svilar et al. [321]. In a reconstituted system, NTHL1 works in concert with XPG to initiate short patch (single-nucleotide) BER [332]. Along these lines, human cells were developed with OGG1 and MUTYH genes mutated (via CRISPR-KO) to block expression and were used to demonstrate that the herbicide paraquat (1,1′-dimethyl, 4,4′-bipyridinium dichloride; PQ) leads to an increase in genome mutations by causing an increase in the base lesion 8-oxoguanine (8OG). OGG1 and MUTYH are DNA glycosylases that remove primarily oxidative stress-induced base lesions such as 8OG, as summarized [321]. Originally characterized by Grollman, Boiteux, and Seeberg in separate reports [333,334,335], the OGG1 enzyme has been extensively characterized for its substrate specificity. Similarly, MUTYH is specific for oxidatively-damaged DNA bases [336,337].

Continued characterization of these and other DNA glycosylases is critical both to further our understanding of their biological roles and to help in the validation and selectivity analysis of DNA glycosylase inhibitors. For example, cells deficient in OGG1 are sensitive to cytarabine [338], and OGG1 functions to repair oxidative lesions with a preference for telomeric sites [339,340]. Inhibitors of OGG1 that can be further clarified for selectivity using CRISPR-KO cells include the O8 compound that exhibits >200-fold selectivity for OGG1 over other glycosylases [341] and the TH5487 compound, reported to be OGG1 specific [342].

Defining the biological role of TDG, the so-named thymine DNA glycosylase, was challenging. Bellacosa et al. demonstrated that the major role for TDG is in active DNA demethylation, with specificity for CpG islands, providing protection from hypermethylation [343]. It was eventually shown that the concerted TET-TDG demethylation/BER pathway plays numerous, critical biological roles. Using targeted CRISPR gene editing approaches, it was shown that loss of TDG promoted the accumulation of 5-formylcytosine, such as that seen in diabetes [344]. The methyl-modification at the C-5 position of cytosine (5 mC) in DNA is a well-studied epigenetic mark and is involved in numerous biological processes. TDG’s role in the demethylation of 5mC involves the ten-eleven translocation (TET) enzymes that carry out the oxidation of 5mC to produce 5-hydroxymethylcytosine (5hmC), 5-formylcytosine (5fC), and 5-carboxylcytosine (5caC) [345,346] yielding the substrate for TDG [347]. More recently, CRISPR-KO of all three TET enzymes revealed a role for this TET-TDG demethylation pathway in telomere-sister chromatid exchange and telomere maintenance [348]. Interestingly, similar gene targeting studies have suggested that both the TET enzymes and TDG may be important clinical targets. The TET2 isoform is suggested to be synthetically lethal with TOP1-targeted drugs or PARP1 inhibitors [349]. Similarly, earlier work has suggested that some cancer types, especially melanoma, may be preferentially sensitive to TDG loss of expression or inhibition [350].

The primary strand scission enzyme in BER is the apurinic/apyrimidinic DNA endonuclease, isoform 1 (APE1, APEX1). Originally identified as a redox regulator of transcription factors, both the DNA repair and redox enzymatic functions of APE1 are overlapping [351,352]. APE1 is the primary AP (apurinic/apyrimidinic) endonuclease in human cells and plays an essential role in BER [201,321,353]. During BER, APE1 functions to hydrolyze such AP or abasic sites in DNA resulting from either spontaneous base loss or from glycosylase-mediated base lesion hydrolysis [201,354,355]. Recent work has also suggested APE1 may play a role in RNA repair [356]. Cells with partial loss of APE1 expression are viable, such as shown in U2OS cells [357]. However, in many cell types, complete loss of APE1 expression is lethal. Some studies have relied on CRISPR-based gene editing (knockout) to uncover unique biological roles for APE1. For example, using CRISPR/Cas9 editing, it was demonstrated that APE1 is a target of the microRNA miR-27a-5p [358]. Further, loss of APE1 in the triple-negative cell line HCC1937 partially suppressed the cell-killing effect of the PARP1 inhibitor, olaparib [359]. These latter studies suggest that APE1 expression may be important for the cellular response to PARP-inhibitors (PARPi) as loss of APE1 reduced the cell-killing effect of the PARPi but not cisplatin.

While APE1 is defined as the primary AP endonuclease for BER and SSBR, defining a biological role for APE2 has been challenging. An early report using *Xenopus* egg extracts suggested a role for APE2 in ATR/CHK1 signaling [360], but how this functioned in human cells was only recently uncovered using CRISPR screening. In several reports, it was found that APE2 is synthetically lethal with BRCA1/BRCA2, suggesting a role for APE2 in the reversal of blocked 3′ DNA ends and TOP1 processing of misincorporation of ribonucleotides [361,362]. The detailed mechanism of APE2-mediated end processing is currently under intense investigation.

#### 6.2.2. DNA Damage-Induced PARP1 and PARP2 Activation in BER/SSBR, in Response to Replication Stress and Advancing Our Understanding of PARP1/PARP2 Inhibitors

PARP1 activation plays a central role in the cellular response to DNA base (BER) and SSB damage (SSBR), in what might be called global BER/SSBR, as well as in the response to replication stress. The activation of PARP1 or PARP2 leads to NAD^+^ hydrolysis and the production of the post-translational modification poly-ADP-ribose (PAR). The modification of PARP1/PARP2 (self-modification) and the surrounding proteins facilitates repair protein complex assembly, a process that is further regulated by cellular NAD^+^ biosynthesis [202,363]. It was noted recently, using complemented PARP1-KO cells, that PARP1 mutants produce variations in chain length and modified chain branching that in turn prompts distinct molecular processes [364]. The activation of PARP1/PARP2 in response to replication stress also recruits the BER/SSBR scaffold protein XRCC1 and so may then be called replication-associated BER/SSBR. The major activator of PARP1 and PARP2 is DNA, specifically single-strand or double-strand DNA breaks [365]. In the context of global BER/SSBR, CRISPR-mediated KO of PARP1 is shown to prevent the formation of BER/SSBR protein complexes at sites of laser-induced DNA damage [366]. Recent studies have also suggested a complicated role for PARP1 in RNA biology, a role for RNA in PARP1 activation, and possibly as a target of PARP1 activation [367,368,369,370,371,372,373]. Further, Krauss et al. suggest the snoRNAs may be endogenous PARP1 activators, confirmed by creating CRISPR/Cas9-mediated KO of snoRNAs in cells [374].

Regarding replication stress, both PARP1 and PARP2 play a redundant role in stabilizing replication forks; each PARP-isoform is suggested to be activated at the fork due to BER intermediates [375]. The chromatin modulator ALC1, described above, appears to promote PARP2 trapping at DNA breaks [318] due to treatment with PARPi. The mechanism of action of many PARPi in cancer treatment involves trapping the target (PARP1/PARP2) in genomic DNA, forming a lethal DNA-protein crosslink [376,377,378]. Using ribonuclease H2-KOs, it was found that ribonucleotides mis-incorporated during replication are considered the primary trapping lesions. The requirement for the expression of PARP1 for the cellular response to PARPi was evaluated by mutating the PARP1 gene via CRISPR/Cas9. Cellular response to PARPi alone and in combination with ATR inhibitors was confirmed [379]. Further, using a novel editing approach, CRISPR-Cas9 “tag-mutate-enrich” mutagenesis screens, Lord et al. show that PARPi resistance can come about from key mutations in the *PARP1* gene [380]. Using a similar KO approach, it was shown that PARP1 is required for ATM-mediated p53 activation in response to ionizing radiation [381]. However, in glioblastoma cells, loss of PARP1 expression by CRISPR-KO did not sensitize MSH6-mutant tumor cells to the alkylating agent temozolomide (TMZ), suggesting a more complicated role for PARP in response to alkylating agents [382].

#### 6.2.3. DNA Gap Tailoring in BER/SSBR

Gap tailoring for BER or SSBR is primarily initiated by PAR-mediated recruitment of the scaffold protein XRCC1 [296], that in turn promotes recruitment of the gap tailoring enzymes Polβ, DNA ligase III, and APTX [201]. As the key protein in the formation of BER/SSBR protein complexes, a significant effort has gone into characterizing human XRCC1-KO cells developed via CRISPR-mediated gene editing. In the absence of XRCC1, Polβ is ubiquitylated, promoting its degradation [383]. Using such XRCC1-KO cells, we also demonstrated that the level of nuclear and chromatin bound Polβ protein is significantly reduced [384]. Further, similar KO cells revealed a role for XRCC1 in response to cisplatin, and it was then demonstrated that transcription partially regulates the chromatin retention of XRCC1 in response to cisplatin exposure, suggesting a possible transcription-coupled BER mechanism [312]. Using XRCC1-KO cells and fluorescently tagged BER proteins, we have shown that XRCC1 is required for the recruitment of Polβ by a mechanism that involves SIRT6 [366]. Similarly, we find that XRCC1 controls the recruitment of most of the BER and SSBR gap tailoring enzymes, including Polβ, DNA ligase III, and APTX, with APLF only dependent on PAR formation and PNKP, which is recruited independently of both PAR and XRCC1 (Manuscript in Preparation). As the major factor that is recruited to sites of PARylation, it has also been shown that loss of XRCC1 leads to elevated and persistent PARP1 activation both for laser-induced DNA damage [366] and in response to replication-stress induced PARP1 activation [385,386,387], the latter at least in part, due to a role for XRCC1 in Okazaki fragment processing [388].

The development of KOs or gene-edited cells for Polβ has not only confirmed its involvement in nuclear BER/SSBR [366], but also in mitochondrial BER [389] and have been used to show that Polβ controls XRCC1 complex dynamics following DNA damage [366] and that Polβ is not required for HIV-1 infection [390]. While Polβ is the predominant DNA polymerase involved in BER, a role for DNA polymerase iota has been suggested [391] due to its 5′dRP lyase activity [392,393,394], its role in response to oxidative stress [395], and repair of clustered base damage [396,397]. However, a possible role for such low fidelity DNA polymerases in BER such as Pols iota, eta, or kappa, has also been disputed [398]. Incidentally, using Pol iota-KO cells, it was confirmed that the loss of Pol iota renders cells sensitive to hydrogen peroxide, supporting its possible role in BER or SSBR [399]. Lig3-KO has been used to reveal an involvement in chromosomal translocations [400], but similar analyses using KO or editing of the *Lig3* gene or the genes of other BER/SSBR gap tailoring enzymes await characterization for enhanced insight into their role in either global or replication-associated BER/SSBR.

#### 6.2.4. PAR Degradation

The dynamics of PAR synthesis and degradation are essential for the timely completion of repair of base damage and SSBs [202,401]. Poly(ADP-ribose) glycohydrolase (PARG)-KO is lethal, but it is clear that blocking PAR degradation that results from replication-stress induced PARP-activation with potent and selective PARG inhibitors leads to activation of an intra S-phase checkpoint via CHK1 activation [385,402]. PAR degradation may also involve TARG1 and ARH3 [403,404,405]. In recent studies, we find that TARG1-KO cells are viable but do not alter BER protein complex dynamics [366]. Further, it is not yet clear if BER or SSBR is impacted by the PARP1/2-binding protein HPF1, found to be essential to mediate PARP1-induced serine-ADP-ribosylation [406,407]. ARH3 maybe be most essential to remove serine ADP-ribosylation [408], but it is not known how this regulator of PARP1/2 (HPF1) influences BER or SSBR protein complex assembly or function. However, using ARH3-KO cells, it was shown that long-term loss of ARH3 results in the accumulation of regions of DNA marked by ADP-ribose, termed ‘ADP-ribose scars’ [409].

## 7. CRISPR Advancements in the Clinical Setting (and Promising Future Advancements)

With the success of CRISPR editing in vitro (cells) and in vivo (animal models), CRISPR technology has now made its way into the clinical setting. As previously mentioned, CRISPR systems have shown success as a diagnostic tool. Currently, clinicaltrials.gov has eight completed, active, or recruiting trials listed globally that involve CRISPR. Beyond diagnostics, the success of CRISPR has propelled this technology into use as a potential therapeutic. There are over 25 completed, active, or recruiting trials across the globe using CRISPR technology for editing outside the body (ex vivo) prior to administration to patients. The ex vivo CRISPR-based immunotherapies primarily utilize chimeric antigen receptor (CAR) T-cell therapy and modified T-cell receptor (TCR) therapy. An overview focusing on the clinical applications of CRISPR/Cas9 can be found here [410]. However, worth mentioning are the three ongoing trials using CRISPR editing systems as a therapy inside the body (in vivo).

Clinical trial NCT03872479 that began in September 2019 is the first of its kind (clinicaltrials.gov) to use an in vivo CRISPR therapeutic. This multi-center (USA-based) trial is evaluating the safety, tolerability, and efficacy of ascending doses of EDIT-101 subretinal injections in patients with Leber Congenital Amaurosis Type 10 (LCA10) [411]. LCA10 is characterized by severe cone-rod dystrophy and poor vision [412]. This rare condition is caused by an autosomal recessive condition due to an IVS26 point mutation (c.2991 + 1655 A > G) within intron 26 of the CEP290 gene, resulting in a splicing defect and early stop codon within the mRNA. EDIT-101 is an AAV5 vector carrying SaCas9 and two highly specific sgRNAs, designed to restore the full-length mRNA and protein via deletion or inversion of intron 26 [411,412]. In preliminary studies using mice and non-human primates, EDIT-101 was well tolerated and showed sufficient editing efficiency for vision restoration, since as little as 10% functional foveal cone photoreceptors are sufficient for near-normal vision [412,413,414]. The trial is estimated to be completed by March 2024.

In November 2020, two new in vivo trials began. The first trial (clinicaltrials.gov; NCT04560790), based in China, is aimed to test the safety, tolerability, and efficacy of single escalating doses of BD111 as a therapy for herpetic stromal keratitis caused by Herpes simplex virus type I (HSV-1) [415]. BD111 is a corneal injection of Cas9 mRNA and sgRNA. The second is a multi-center trial (clinicaltrials.gov; NCT04601051) in New Zealand and the UK for an in vivo CRISPR therapeutic (NTLA-2001) in the treatment of hereditary transthyretin amyloidosis with polyneuropathy (ATTRv-PN) [416]. Hereditary ATTR is caused by mutations within the *TTR* gene that result in misfolding of the protein transthyretin (TTR). The misfolded TTR aggregates into amyloid fibrils in various organs resulting in dysfunction [417]. NTLA-2001 consists of Cas9 mRNA and sgRNA delivered by lipid nanoparticles intravenously, making it the first in vivo CRISPR therapy to be administered systemically [418,419]. As a Phase 1 trial, the aim is to evaluate the safety, tolerability, pharmacokinetics, and pharmacodynamics of NTLA-2001 in ATTRv-PN patients to determine the optimal biologically active dose [416]. The trial is set to be completed by March 2024.

## 8. Conclusions

For decades, the ‘awesome power of yeast genetics’ has allowed the rapid analysis of yeast gene mutations to uncover the biological roles of many genes and gene pathways under diverse cellular conditions [420,421]. While RNAi brought us one step closer to a facile toolset for gene suppression [422], the advent of CRISPR/Cas9 editing systems has rapidly brought biochemistry-level genetic manipulation to the mammalian cell, opening the door to in vivo and cell-based analysis of almost any gene, gene mutation, or the capacity to evaluate the repercussions of complete gain or loss. Clearly, CRISPR has revolutionized the way we think about conducting research. The impressive body of literature that has resulted from the advent of CRISPR technology has impacted many, if not all, facets of cellular biology. In this article, we present a small snapshot of the utility of gene editing tools that can be brought to bear to further our knowledge of two DNA repair pathways, MMR and BER. The far-reaching implications of CRISPR-based gene editing go well beyond basic science research with roles in medicine and therapeutics [423], gene therapy [424], agriculture [425], advances in animal modeling [426], and cancer discovery [427]. CRISPR methods open the door to the personalized treatment of genetic diseases. Ongoing clinical trials utilizing CRISPR technologies hold the potential to revolutionize the future of medicine.

## Figures and Tables

**Table 1:** Single subunit Cas endonucleases and their fun facts.

Cas Ortholog	Size (aa)	Origin	Engineered Variant	Mutation	Target Substrate	Trans-Cleavage	PAM/PFS 5′-3′	PAM Frequency Compared to spCas9 ^1^	Feature	Refs
SpCas9	1368	*Streptococcus pyogenes*	-	-	dsDNA	-	NGG	1.00	Most commonly used	[79,80]
			nCas9	D10A or H840A	dsDNA	-	NGG	1.00	Only one active nuclease domain	[26]
			dCas9	D10A/H840A	dsDNA	-	NGG	1.00	Nuclease deficient	[26]
			xCas9 3.7	A262T/R324L/S409I/E480K/E543D/M694I/E1219V	dsDNA	-	NGN, GAA, GAT	3.12	Broadened PAM compatibility	[81,82]
			HypaCas9	N692A/M694A/Q695A/H698A	dsDNA	-	NGG	1.00	Hyper-accurate, increased fidelity	[83]
			Cas9 D1135E	D1135E	dsDNA	-	NGG	1.00	Reduced binding to non-canonical PAM (NAG)	[84]
			Cas9 VQR	D1135V/R1335Q/T1337R	dsDNA	-	NGAN, NGNG	1.59	Altered PAM	[84]
			Cas9 VRER	D1135V/G1218R/R1335E/T1337R	dsDNA	-	NGCG	0.04	Altered PAM	[84]
			Cas9 EQR	D1135E/R1335Q/T1337R	dsDNA	-	NGAG	0.37	Altered PAM	[84]
			eCas9 1.1	K848A/K1003A/R1060A	dsDNA	-	NGG	1.00	Refined DNA-NUC lobe contact, enhanced specificity	[85]
			Cas9-HF1	N497A/R661A/Q695A/Q926A	dsDNA	-	NGG	1.00	High fidelity, refined DNA-REC lobe contact, reduce non-specific interactions	[86]
			HifiCas9	R691A	dsDNA	-	NGG	1.00	Designed for RNP delivery	[87]
NmCas9	1082	*Neisseria meningitides*	-	-	dsDNA	-	NNNNGATT	0.10	Reduced target range and restricted off-target sites	[88]
SaCas9	1053	*Staphylococcus aureus*	-	-	dsDNA	-	NNGRRT	0.34	Alternative PAM, smaller Cas protein	[89]
			Cas9 KKH	E72K/N968K/R1015H	dsDNA	-	NNNRRT	1.09	Altered PAM	[90]
CjCas9	984	*Campylobacter jejuni*	-	-	dsDNA ssRNA	-	NNNVRYM	1.11	Smallest Cas9	[91]
FnCas9	1629	*Francisella novicida*	-	-	dsDNAssRNA	-	NGG	1.00	Can cleave ssRNA	[43,92]
			FnCas9 RHA	E1369R/E1449H/R1556A	dsDNA	-	YG	2.00	Shortest Cas9 PAM	[43,92]
St1Cas9	1121	*Streptococcus thermophilius*	-	-	dsDNA	-	NNAGAAW	0.11	Alternative PAM	[93]
St3Cas9	1409		-	-	dsDNA	-	NGGNG	0.26	Alternative PAM	[94]
TdCas9	1395	*Treponema denticola*	-	-	dsDNA	-	NAAAAC	0.04	A-rich PAM	[93]
AsCas12a (Cpf1)	1307	Acidaminococcus sp. BV3L6	-	-	DNA	ssDNA	TTTN	0.50	Only uses crRNA	[63,95,96]
FnCas12a (Cpf1)	1300	*Francisella novicida*	-	-	DNA	ssDNA	TTN	1.45	Only uses crRNA	[63,95,96]
AacCas12b (C2c1)	1277	*Alicyclobacillus acidoterrestris*	-	-	DNA	ssDNA	TTN	1.45	PAM at 5′ end, high specificity, requires tracrRNA	[13,66,97]
OspCas12c (C2c3)	1252	Oleiphilus sp.	-	-	DNA	ssDNA	TG	1.88	Requires scoutRNA for crRNA processing	[98,99]
Cas12d (CasY)	1200	Uncultivated	-	-	DNA	ssDNA	TA	1.38	Requires scoutRNA and crRNA for target cleavage	[99,100]
Cas12e (CasX)	980	Deltaproteobacteria	-	-	dsDNA	ssDNA	TTCN	0.49	Alternative PAM, smaller Cas12, requires scoutRNA for crRNA maturation	[70,99,100]
Cas12f (Cas14)	400–700	Uncultivated	-	-	ssDNA	ssDNA, dsDNA	TTTR, TTAT	0.40	Smallest known Cas, longer sgRNA, non-specific cleavage of ssDNA, requires tracrRNA	[26,101,102,103]
Cas12g	767	Uncultivated	-	-	ssRNA	ssRNA, ssDNA	No Requirement	-	Does not target dsDNA	[98,100,101]
Cas12h	870	Uncultivated	-	-	DNA	ssDNA	RTR	1.17	Alternative PAM	[98]
Cas12i	1093	*Lachnospiraceae bacterium*	-	-	DNA	ssDNA	TTN	1.45	Predominantly a nickase cutting the NTS, requires a longer crRNA spacer pairing for TS cleavage	[72,98]
Cas12j (Casφ)	~750	*Biggiephage*	-	-	DNA	ssDNA	TBN	2.93	Smaller sgRNA, discovered in bacterial phages	[104]
Cas12k	~650	*Scytonema hofmanni*	-	-	dsDNA	-	GTN	1.17	Requires tracrRNA	[105]
LshCas13a (C2c2)	1389	*Leptotrichia shahii*	-	-	ssRNA	ssRNA	H	-	RNA targeting, two HEPN domains, only uses crRNA, cleaves outside the base-pairing region	[13,97,106,107]
LwaCas13a (C2c2)	1152	*Leptotrichia wadei*	dCas13a	R474A/R1046A	ssRNA	ssRNA	No requirement	-	Nuclease deficient RNA binding protein	[108]
BzCas13b (C2c6)	1224	*Bergeyella zoohelcum*	-	-	ssRNA	ssRNA	D-(PS)-NAN/NNA	-	Recognizes bases on either side of the protospacer	[109]
EsCas13d	954	*Eubacterium siraeum*	-	-	ssRNA	ssRNA	No requirement	-	Smaller than other Type VI subtypes	[68,110]

Abbreviations: PS = Protospacer; B = G, C, or T; N = T, C, G, or A; Y = C or T; W = A or T; R = A or G; V = A, C, or G; M = A or C; H = A, T/U, or C; D = A, G, or T; NTS = non-target strand; TS = target strand. ^1^ Frequency is based on occurrence of the PAM sequence and its reverse complement in the GRCh38 genome (GCF_000001405.39). Values > 1 indicate a higher occurrence than the spCas9 PAM. Values < 1 indicate PAMs that occur less frequently.

## Data Availability

The python script used to generate the PAM frequency data included in Table 1 is available online: https://github.com/mkmellon5/MCI (accessed on 29 March 2021).
